# Tracking socio-economic inequalities in healthcare utilization in Iran: a repeated cross-sectional analysis

**DOI:** 10.1186/s12889-020-09001-z

**Published:** 2020-06-15

**Authors:** Sajad Vahedi, Vahid Yazdi-Feyzabadi, Mostafa Amini-Rarani, Abolfazl Mohammadbeigi, Ardeshir Khosravi, Aziz Rezapour

**Affiliations:** 1 Department of Healthcare Administration, School of Health, Ahvaz Jundishapur University of Medical Sciences, Ahvaz, Iran; 2 Health Services Management Research Center, Institute for Futures Studies in Health, Kerman University of Medical Sciences, Kerman, Iran; 3 Health Management and Economics Research Center, Isfahan University of Medical Sciences, Isfahan, Iran; 4 Neuroscience Research Center, Department of Epidemiology and Biostatistics, Faculty of Health, Qom University of Medical Sciences, Qom, Iran; 5 Deputy for Public Health, Ministry of Health and Medical Education, Tehran, Iran; 6 Health Management and Economics Research Center, School of Health Management and Information Sciences, Iran University of Medical Sciences, P.O Box: 1996713883, No. 6, Rashid Yasemi St. Vali –e Asr Ave, Tehran, Iran

**Keywords:** Iran, Health inequality, Healthcare services, Concentration index, Decomposition analysis

## Abstract

**Background:**

Although some healthcare reforms such as Health Transformation Plan (HTP) were implemented in Iran to provide required healthcare services, few studies have been conducted to track the impacts of these reforms on socio-economic inequality in healthcare utilization. This study aims to track socio-economic inequalities in healthcare utilization and their changes between 2008 and 2016 in Iran.

**Methods:**

Required data were obtained from two of Iran’s utilization of healthcare services survey conducted in 2008 and 2016. Erreygers concentration index (EI) was used to measure inequality in the utilization of outpatient and inpatient healthcare services (UOH and UIH). The decomposition of EI (DEI) was used to explain healthcare utilization inequality. Oaxaca decomposition (OD) was also employed to track the changes in EI in this period.

**Result:**

Inequality in UOH increased from 0.105 to 0.133 in the studied years, indicating the pro-rich distribution of UOH. Inequality in UIH decreased from 0.0558 to − 0.006. DEI showed that economic status was the main factor that contributed to inequality in the UOH and UIH. OD showed that residence in rural areas and supplementary insurance were the main contributing factors in the increased inequality of UOH. Moreover, OD also showed that economic status was the main contributing factor in the reduced inequality of UIH.

**Conclusion:**

While Iran still suffers from significant socio-economic inequalities in UOH, it seems that healthcare reforms, especially HTP, have reduced UIH inequality. Expanding healthcare reforms into the outpatient sector and also implementing effective health financing policies could be recommended as a remedy against UOH inequality.

## Background

Equitable utilization of healthcare services for those in need without suffering from financial hardship has been emphasized in the universal health coverage (UHC) paradigm [1–3]. This matter has also been re-emphasized in Sustainable Development Goals to ensure healthy lives and well-being promotion for all and at all ages [4]. Healthcare policymakers strongly recommend equal treatment for equal needs for healthcare services, regardless of the socio-economic and cultural background that lead to the horizontal equity concept [5, 6].

Worldwide, governments are under intense political pressures to increase their spending to provide equal access to healthcare services [7]. Despite the increase in public health expenditure, evidence showed that socioeconomic inequality in the utilization of required healthcare services is still persistent. Unequal access to these services could deteriorate inequality in health outcomes and increase morbidity and mortality, especially among disadvantaged groups [8]. These inequalities are not restricted to less-developed countries [9] but have also been reported from developed countries in Europe [10], America [11], and Asia [12]. Tracking health inequalities and trying to explain the factors affecting them are crucial to the health systems to boost their performance [13].

Iran is an upper-middle-income country located in the Eastern Mediterranean region. According to the latest census of Iran, this country had approximately 80 million people that living across 31 provinces. Iran almost spends 7% of its gross domestic product in the healthcare sector [7]. The concept of equitable access to equitable needs is reflected in the high-level national documents of Iran, such as the Constitution of the Islamic Republic of Iran and 5-year economic development programs [8]. Iran has primarily an insurance-based healthcare system [14] that provides healthcare services through public, non-governmental organizations, and an extensive network of private providers [15]. While the public sector of Iran has been active in all levels, this sector is the only provider in the first level through providing primary healthcare services. The private sector of Iran, alongside the public sector, mainly provides secondary and tertiary healthcare services [16]. Ministry of Health and Medical Education (MOHME) predominantly provides inpatient healthcare services through ownership of the majority of hospital beds. The private sector and social security organization are the other essential providers of inpatient healthcare services in Iran [17]. After the revolution, Iran implemented numerous healthcare reforms and policies, such as the integration of provincial health organizations within medical sciences universities, the establishment of primary health care network and Universal Health Insurance Act to provide required healthcare services and boost health equity [18]. In adition, trying to activate the public sector, Iran raised per capita government’s spending on health from 171.6 up to 673.6 dollars between 2000 and 2015 [19]. Although these programs increase the access to basic healthcare services, considerable inequality in the utilization of healthcare services and health outcomes have been reported in the national [16] and sub-national levels [20] of Iran. Recently, Iran introduced an UHC program in 2014 entitled the healthcare transformation plan (HTP) to ensure the availability and accessibility of required healthcare services for all [8]. More details regarding the HTP have been published elsewhere [21]. The main purpose of the current study was to measure and explain potential inequality in the utilization of healthcare services in Iran and try to track and explain its change across time. Measuring the inequality in different periods could provide valuable evidence for policy makers and the society as a whole to know the extent to which inequality exists, how inequalities change over time in an array of applied policies and the factors affecting the reduction or expansion of inequalities.

## Methods

### Study setting and data

The required data for this repeated cross-sectional study was obtained from the first and last waves of a national survey titled the Utilization of Health Services in IR Iran (IrUHSS). The purpose of IrUHSS was to gather and prepare national and sub-national information about the status of the utilization of healthcare services and the impacts of demographic and socioeconomic status on it. The MOHME conducted the first wave of this survey in 2008. The last survey was ordered by MOHME and conducted via the collaboration of the National Institute of Health Research and Statistical Center of Iran in 2016. Multi-stage proportional stratified cluster sampling was used in IrUHSS. Because of the difference in the population size of Iranian provinces and their districts, the proportion of each district was determined from the total sample size. Subsequently, random samples of clusters in each district were selected and weighted according to the rural and urban populations within each region. IrUHSS used a pre-tested and validated questionnaires, which included questions about household demographics, education, and durable assets, healthcare services need, and care-seeking behavior. To avoid the missing data bias and potential recall bias, the analysis was limited to people above 15 years of age.

### Measuring healthcare services utilization and definition of variable

Several questions of IrUHSS were used in this study to measure the need for healthcare and the utilization of healthcare services. Two measures of outpatient and inpatient healthcare service needs were estimated using two different questions on IrUHSS: “did you have any outpatient health care services need during the last two weeks?” and “did you have any inpatient healthcare services need during the last year?” The question “have you received any outpatient services during the last two weeks?” in the IrUHSS is used to compute the probabilities of the utilization of the outpatient healthcare services (UOH). On the other hand, the question “have you received any inpatient services during the last two weeks?” is used to measure inpatient healthcare utilization (UIH).

In this study, ten age-sex groups alongside the number of outpatient and inpatient healthcare needs were included in the analysis as need factors. On the other hand, the place of residence (urban/rural), marital status (married/unmarried), employment status (employed/unemployed), education (illiterate/primary/ secondary/ diploma/higher), basic health insurance (no health insurance/ Iranian/Social security/other insurance) and supplementary insurance (Yes/NO) were considered as non-need factors. The health system in Iran is largely funded by government revenue, public and private health insurance schemes, and out-of-pocket payments. The most important public insurance organizations of Iran are as follows: Iran Health Insurance Organization (IHIO), Social Security Organization (SSO), Army Medical Insurance Organization, and Imam Khomeini Relief Foundation Health Insurance. There were other entities such as the Ministry of Petroleum, the banking system, and the Municipality of greater Tehran that may provide basic insurance programs for their employees. In addition, there are private insurance programs that offset the co-payment of health expenses and offer other services that are not provided by the basic health insurance schemes [22]. The IrUHSS asked the participant about coverage of basic insurance programs and supplementary (private) insurance. As the majority of participants covered by IHIO and SSO, we categorized the basic insurance into Iranian, Social security, and other insurance programs. Furthermore, the wealth score of the studied participants was regarded as economic status.

There are no accurate data about income and expenditure in the IrUHSS. Hence, the principal component analysis (PCA) was used to construct the economic status of the participants by using household assets. PCA has been widely used in previous studies to measure socioeconomic status [3, 8, 23–25]. Two classes of variables, including the characteristic of housing (e.g., house ownership and floor area) and possession of assets (e.g., private car, motorcycle, personal computer, Internet access, kitchen, telephone, and central heating machine), were used to construct the wealth score. Finally, the constructed wealth score was divided into five quintiles (i.e., poorest, poor, middle, rich, and richest) to be used in the subsequent analyses.

### Inequality analysis

The concentration index (CI) was employed to show the degree of inequality in healthcare utilization. CI equals twice the area between the concentration curve and the line of equality [26] and could be obtained from the covariance between the healthcare utilization and the fractional rank of the individual sorted by economic status:

1 $$ CI(y)=\frac{2}{\overline{y}} Cov\ \left({y}_i.{R}_i\right) $$

Where *y*_*i*_ is the dummy variable of whether the *i*_*th*_ person has utilized healthcare services or benefited from out/inpatient services in the last 2 weeks/1 year, $\overline{y}$ denotes the mean of healthcare utilization, *R*_*i*_ stand for the fractional rank of the *i*_*th*_ individual by economic status and Cov is the covariance with sampling weights. Positive values of CI indicate that the utilization of healthcare services is concentrated among the wealthier and vice versa.

As the outcome of interest in this study is a binary variable, the corrected concentration index suggested by Erreygers [27] was used. Erreygers concentration index (EI) could be defined as flow:

2 $$ EI(y)=\frac{4\overline{y}}{\Big({y}_{max}-{y}_{\min \Big)}} CI(y) $$

Where *y*_*max*_ and *y*_*min*_ show the maximum and minimum of healthcare utilization, and *CI* (*y*) is obtained from eq. (1).

### Decomposition of concentration index and measuring horizontal equity

The regression-based decomposition analysis was used to assess the extent to which each determinant factor contributed to the inequality in healthcare utilization [28]. Accordingly, it is required that the coefficients of determinant factors in the regression analysis be included in the decomposition analysis. As the nonlinear regression model was proposed for the decomposition of binary outcomes, the generalized linear model (GLM) with an identity link function was run to obtain the regression coefficients of explanatory variables. Furthermore, the decomposition analysis was conducted using eq. (3) to getting coefficients of regressors and eq. (4) to determine absolute contributions [29].

3 $$ y={\beta}_0+{\sum}_j{\beta}_j{x}_{ij}+{\sum}_k{\delta}_k{z}_{ik}+{\varepsilon}_i $$

4 $$ {EI}_y=\sum \limits_i{\beta}_j{EI}_j+\sum \limits_j{\delta}_k{EI}_k+ GCE $$

In equation (4), *i* denotes the *i* th individual, *x*_*ij*_ refers to the *j* th need factor of the *i* th individual, *z*_*ik*_ is the *k* th non-need factor and economic status; *β*_*j*_ and *δ*_*k*_ are coefficient of included regressors. In equation (4), *β*_*j*_*EI*_*j*_ and *δ*_*k*_*EI*_*k*_ stand for the absolute contributions of determinant factors, and GCE shows a generalized concentration index of the error term. A positive (negative) contribution indicates that the given determinant factor operates towards the pro-rich (pro-poor) distribution of healthcare utilization. Following Wagstaff, the degree of horizontal inequity could be obtained through subtracting the absolute contribution of need factors from EI of healthcare utilization. Finally, the change in EI of healthcare utilization from 2008 to 2016 was decomposed using the Oaxaca-like decomposition to estimate the contribution of the change in determinants’ inequality and the change of their marginal effects. Hence, the decomposition of the change in EI can be written as equation (5):

5 $$ \Delta  {EI}_y=\sum {\beta}_{(2008)}\left(\Delta  EI\left({x}_i\right)\right)+\sum {EI}_{(2016)}\left(\Delta  \beta \right)+\Delta  GCE $$

Were *β*_(2008)_(*∆EI*(*x*_*i*_)) shows an absolute change of inequality of regressors, and *EI*_(2016)_(*∆β*) stands for the absolute change of their marginal effect. All analyses were conducted in STATA 12/SE. In addition, SigmaPlot 12.0 was used to generate the figures representing the decomposition of inequality in healthcare utilization.

## Results

### The status of healthcare utilization and its inequality

Table 1 shows the descriptive statistics of UOH and UIH and their determinants across time. While the mean of UOH decreased from 0.671 to 0.629 after 2016, the level of UIH increased from 0.811 to 0.920.

**Table 1:** Sample characteristics by utilization of outpatient and inpatient healthcare service use, Iran

Variable	Utilization of outpatient				Utilization of inpatient			
	2008		2016		2008		2016	
	N	%	N	%	N	%	N	%
Total sample	18,515	100	13,005	100	7149	100	4864	100
Healthcare utilization	12,431	67.14	8181	62.91	5799	81.12	4479	92.08
**Need factors**								
Sex-age groups								
Male 15–29	2384	12.88	1170	9.00	944	13.20	332	6.83
Male 30–44	2136	11.54	1411	10.85	681	9.53	434	8.92
Male 45–59	1532	8.27	1247	9.59	652	9.12	478	9.83
Male 60–74	1007	5.44	930	7.15	589	8.24	445	9.15
Male 75≤	582	3.14	452	3.48	363	5.08	239	4.91
Female 15–29	3465	18.71	1725	13.26	1274	17.82	799	16.43
Female 30–44	3212	17.35	2342	18.01	1126	15.75	926	19.04
Female 45–59	2436	13.16	2068	15.90	793	11.09	601	12.36
Female 60–74	1347	7.28	1298	9.98	515	7.20	455	9.35
Female 75≤	414	2.24	362	2.78	212	2.97	155	3.19
Healthcare needs								
One healthcare need (Need1)	13,219	71.40	10,420	80.12	6028	84.32	4527	93.07
Two healthcare need or higher (Need2)	5296	28.60	2585	19.88	1121	15.68	337	6.93
**Non-need factors**								
Residence								
Urban	9135	49.34	8698	66.88	3550	49.66	3294	67.72
Rural	9380	50.66	4307	33.12	3599	50.34	1570	32.28
Marital status								
Married	13,445	72.62	9720	74.74	5443	76.14	3935	80.9
Unmarried	5070	27.38	3285	25.26	1706	23.86	929	19.1
Employment status								
Employed	5362	28.96	3101	23.84	2039	28.52	1006	20.68
Unemployed	13,153	71.04	9904	76.16	5110	71.48	3858	79.32
Education								
Illiterate	5712	30.85	3446	26.50	2420	33.85	1288	26.48
Primary	6175	33.35	3353	25.78	2333	32.63	1236	25.41
Secondary	2678	14.46	2104	16.18	961	13.44	769	15.81
Diploma	2497	13.49	2289	17.60	959	13.41	908	18.67
Higher	1453	7.85	1813	13.94	476	6.66	663	13.63
Basic insurance								
No insurance	2729	14.74	792	6.09	908	12.7	206	4.24
Iranian	9952	53.75	5888	45.27	3946	55.2	2143	44.06
Social	4511	24.36	5155	39.64	1724	24.12	2022	41.57
Other	1323	7.15	1170	9	571	7.99	493	10.14
Supplementary insurance								
Yes	2090	11.29	2455	18.88	775	10.84	1124	23.11
No	16,425	88.71	10,550	81.12	6374	89.16	3740	76.89
**Economic status**								
Wealth quintiles								
Q1 (poorest)	3705	20.01	2630	20.22	1434	20.06	946	19.45
Q2	3707	20.02	2583	19.86	1424	19.92	1011	20.79
Q3	3704	20.01	2591	19.92	1431	20.02	945	19.43
Q4	3697	19.97	2600	19.99	1430	20.00	987	20.29
Q5(richest)	3702	19.99	2601	20.00	1430	20.00	975	20.05

Table 2 represents the socio-economic inequality in UOH and UIH. There was a positive EI of UOH in both years, indicating that the utilization of these services had pro-rich distribution. The EI of UOH significantly (p-value =0.022) increased from 0.105 (95%CI: 0.089, 0.121) in 2008 to 0.133 (95%CI: 0.115, 0.151) in 2016. During this period, the EI of UIH, indicating that the pro-rich healthcare utilization in 2008 significantly (p-value =0.000), decreased from 0.055 (95%CI: 0.035, 0.071) to − 0.006 (95%CI: − 0.022, 0.01) in 2016 and changed its profile from pro-rich to pro-poor.

**Table 2:** Socioeconomic inequality in outpatient and inpatient healthcare utilization before and after the health transformation plan in Iran and their changes

Healthcare utilization	2008			2016			Change over time		
	EI	95%CI	p-value	EI	95%CI	p-value	EI	95%CI	p-value
Outpatient	0.105	0.089, 0.121	0.000	0.133	0.115, 0.151	0.000	0.028	0.004, 0.052	0.022
Inpatient	0.055	0.035, 0.071	0.000	−0.006	− 0.022, 0.01	0.456	− 0.062	− 0.09, − 0.034	0.000

*CI* Confidence interval

### Decomposition of socioeconomic inequalities in healthcare utilization and their changes

The results of the decomposition of inequality in UOH and UIH are demonstrated in Tables 3 and 4, respectively. These tables show the regression coefficients (β) obtained by estimating the GLM model, EI of independent variables, absolute contribution (obtained from the multiplication of coefficient), EI of explanatory variables, and percentage contributions. The last columns of these tables are assigned to Oaxaca decomposition. Similar to the EI of healthcare utilization, the EI of explanatory variables can help inform their distribution regarding socioeconomic status. For example, the EI of Need 2 was negative, indicating that this variable was dominantly concentrated among the poor.

**Table 3:** Decomposition of Erreygers Concentration Index (EI) of outpatient healthcare utilization before and after health transformation plan, and of its change, Iran

	2008				2016				Change			
	β	EI	Absolute Contr.	% Contr.	β	EI	Absolute Contr.	% Contr.	*∆EI*. *β*_2008_	*∆β*. *EI*_2016_	Absolute Contr.	% Contr.
**Need factors**												
Sex-age groups												
Male 30–44	− 0.022	0.023	− 0.001	− 0.48	− 0.123*	0.012	− 0.002	−1.14	0.000	− 0.001	− 0.001	−3.58
Male 45–59	0.015	0.054	0.001	0.78	− 0.046	0.054	− 0.003	−1.88	0.000	− 0.003	− 0.003	− 11.75
Male 60–74	0.059^**^	− 0.020	− 0.001	−1.14	− 0.001	−0.020	0.000	0.02	0.000	0.001	0.001	4.30
Male 75≤	0.116^*^	−0.036	−0.004	−3.93	0.016	−0.040	− 0.001	− 0.46	0.000	0.004	0.004	12.39
Female 15–29	0.056^*^	−0.004	0.000	−0.24	0.016	0.009	0.000	0.11	0.001	0.000	0.000	1.39
Female 30–44	0.078^*^	0.016	0.001	1.20	−0.022	0.042	− 0.001	− 0.70	0.002	− 0.004	− 0.002	−7.76
Female 45–59	0.055^**^	0.022	0.001	1.15	0.001	0.040	0.000	0.03	0.001	−0.002	− 0.001	−4.15
Female 60–74	0.118^*^	−0.049	− 0.006	−5.52	0.023	− 0.082	− 0.001	−1.40	− 0.004	0.008	0.004	13.85
Female 75≤	0.110^*^	−0.026	−0.003	−2.70	0.029	−0.044	− 0.002	− 0.94	− 0.002	0.004	0.002	5.61
Healthcare needs												
Need2	−0.152^*^	− 0.069	0.010	9.97	−0.121	−0.092	0.011	8.37	0.003	−0.003	0.001	2.41
**Non-need factors**												
Residence												
Rural	0.017	−0.530	− 0.009	−8.65	− 0.031*	− 0.367	0.012	8.66	0.003	0.018	0.021	72.88
Marital status												
Married	0.046^*^	0.043	0.002	1.87	0.065*	0.046	0.003	2.23	0.000	0.001	0.001	3.56
Employment status												
Employed	−0.041^*^	0.081	−0.003	−3.12	− 0.022	0.086	− 0.002	− 1.44	0.000	0.002	0.001	4.79
Education												
Illiterate	−0.030	− 0.360	0.011	10.23	−0.045**	− 0.350	− 0.002	11.69	0.000	0.005	0.005	17.11
Primary	−0.005	− 0.114	0.001	0.56	−0.019	−0.132	0.016	1.89	0.000	0.002	0.002	6.83
Secondary	−0.042^**^	0.070	−0.003	−2.83	0.007	0.015	0.003	0.08	0.002	0.001	0.003	10.86
Diploma	−0.007	0.207	−0.002	−1.47	0.001	0.168	0.000	0.07	0.000	0.001	0.002	5.76
Basic insurance												
Iranian	0.077^*^	−0.363	− 0.028	−26.57	0.047**	−0.253	−0.012	−8.85	0.008	0.008	0.016	56.88
Social	0.109^*^	0.256	0.028	26.52	0.097*	0.261	0.025	19.02	0.001	−0.003	− 0.002	−8.80
Other	0.121^*^	0.071	0.009	8.13	0.086*	0.029	0.002	1.86	−0.005	− 0.001	− 0.006	−21.41
Supplementary insurance												
Yes	−0.017	0.203	−0.004	−3.36	0.057*	0.261	0.015	11.18	−0.001	0.019	0.018	65.13
**Economic status**												
Wealth quintiles												
Q2	0.055^*^	−0.341	− 0.019	−17.82	0.031**	−0.340	− 0.011	−8.01	0.000	0.008	0.008	28.37
Q3	0.086^*^	−0.079	− 0.007	−6.45	0.060*	− 0.058	− 0.003	−2.60	0.002	0.002	0.003	11.67
Q4	0.105^*^	0.264	0.028	26.29	0.050*	0.271	0.014	10.27	0.001	−0.015	−0.014	−49.17
Q5	0.133^*^	0.728	0.097	92.35	0.093*	0.714	0.066	49.58	− 0.002	−0.029	− 0.031	−109.12
**Total observed**			0.084	94.80			0.131	97.61			0.031	108.05
**Residual**			0.021	5.20			0.003	2.39			−0.002	−8.05
**Contribution of Need factors**			−0.001				0.003					
**Horizontal inequity**			0.106				0.131					

*: *p* < 0.01, **: *p* < 0.05

**Table 4:** Decomposition of Erreygers Concentration Index (EI) of inpatient healthcare utilization before and after health transformation plan, and of its change, Iran

	2008				2016				Change			
	β	EI	Absolute Contr.	% Contr.	β	EI	Absolute Contr.	% Contr.	*∆EI*. *β*_2008_	*∆β*. *EI*_2016_	Absolute Contr.	% Contr.
**Need factors**												
Sex-age groups												
Male 30–44	− 0.116^*^	− 0.003	0.000	0.54	− 0.070*	0.012	− 0.001	12.00	−0.002	0.001	−0.001	1.80
Male 45–59	−0.062^**^	0.036	−0.002	−4.05	− 0.041	0.075	−0.003	44.76	−0.002	0.002	−0.001	1.30
Male 60–74	0.006	−0.014	0.000	−0.14	− 0.010	0.009	0.000	1.34	0.000	0.000	0.000	0.03
Male 75≤	0.027	−0.046	−0.001	−2.25	0.004	−0.041	0.000	2.09	0.000	0.001	0.001	−1.78
Female 15–29	0.019	0.017	0.000	0.59	0.020	−0.055	−0.001	15.88	−0.001	0.000	−0.001	2.26
Female 30–44	−0.013	0.035	0.000	−0.83	0.009	0.045	0.000	−5.64	0.000	0.001	0.001	−1.36
Female 45–59	−0.051	0.040	−0.002	−3.61	− 0.032	0.052	− 0.002	24.10	− 0.001	0.001	0.000	−0.58
Female 60–74	0.024	−0.035	−0.001	−1.51	0.002	−0.064	0.000	1.78	−0.001	0.001	0.001	−1.15
Female 75≤	0.105^*^	−0.023	−0.002	−4.39	0.030	−0.040	− 0.001	17.56	− 0.002	0.003	0.001	−1.99
Healthcare needs												
Need2	−0.086^*^	−0.054	0.005	8.25	0.006	−0.016	0.000	1.38	−0.003	−0.001	− 0.005	7.50
**Non-need factors**												
Residence												
Rural	−0.004	−0.005	0.002	3.57	0.018	−0.016	−0.006	93.50	−0.001	− 0.008	−0.008	13.43
Marital status												
Married	0.044^*^	0.001	0.001	2.65	0.029	0.054	0.002	−22.81	0.001	−0.001	0.000	−0.14
Employment status												
Employed	0.015	0.042	0.001	1.11	0.005	0.089	0.000	−6.69	0.001	−0.001	0.000	0.25
Education												
Illiterate	−0.083^*^	−0.341	− 0.022	50.43	− 0.030	−0.286	0.006	−92.22	−0.005	− 0.017	−0.022	34.79
Primary	−0.075^*^	−0.074	− 0.012	9.99	− 0.016	−0.140	0.002	−24.33	0.005	−0.009	−0.004	6.23
Secondary	−0.070^*^	0.044	0.006	−5.48	0.001	−0.034	−0.000	2.83	0.005	−0.003	0.003	−4.57
Diploma	−0.037	0.216	0.007	−14.33	0.017	0.169	0.001	−18.34	0.002	0.008	0.009	−14.77
Basic insurance												
Iranian	0.101	−0.352	−0.035	−63.37	−0.029	− 0.266	0.008	− 113.67	0.009	0.035	0.043	−68.89
Social	0.101^*^	0.240	0.024	43.38	−0.015	0.265	−0.004	57.38	0.003	−0.031	−0.028	44.92
Other	0.097	0.07^*^9	0.008	13.69	−0.030	0.023	−0.001	10.05	−0.005	−0.003	− 0.008	13.29
Supplementary insurance												
Yes	−0.024	0.161	−0.004	−6.98	0.025*	0.283	0.007	−105.78	−0.003	0.014	0.011	−17.81
**Economic status**												
Wealth quintiles												
Q2	0.041^**^	−0.333	−0.014	−24.26	0.009	−0.355	−0.003	44.63	−0.001	0.011	0.010	−16.70
Q3	0.018	−0.072	−0.001	−2.29	− 0.001	−0.048	0.000	−0.78	0.000	0.001	0.001	−2.13
Q4	0.056^*^	0.274	0.015	27.48	−0.031*	0.271	−0.008	121.45	0.000	−0.024	−0.024	37.78
Q5	0.049^*^	0.716	0.035	62.40	−0.005	0.714	−0.004	53.14	0.000	−0.038	−0.039	61.39
**Total observed**			0.067	90.59			−0.007	113.59			−0.058	93.11
**Residual**			−0.011	9.41			0.000	−13.59			−0.004	6.89
**Contribution of Need factors**			−0.004				−0.008					
**Horizontal inequity**			0.060				0.001					

*: *p* < 0.01, **: *p* < 0.05

The result of GLM revealed that among the need variables, need2 significantly decreased the probability of UOH in both periods. On the other hand, this variable had a negative association with UIH only in 2008. Married participants had a significant positive association with UOH for both studied years, but this association for UIH only observed in 2008. While residence in rural areas had no significant relationship with UIH, rural participants had a significant negative correlation with UOH in 2016. Different basic insurance programs increased healthcare utilization in 2008 but had no significant effects with UIH in 2016. Moreover, having supplementary insurance increased healthcare utilization only in 2016. Compared to the poorest quintile, while upper economic quintiles had a positive association with healthcare utilization in 2008 and UOH in 2016, these quintiles had a negative association with UIH in 2016 that was significant only for forth quintile. Detailed regression analyses are presented in Tables 3 and 4.

Tables 3 and 4 also provide absolute and percentage contributions of explanatory variables to inequality in UOH and UIH, respectively. Economic status was the main driver of inequality in the UOH and UIH in both years. While this factor explained more than 100% of the inequality of UOH before in 2008, its contribution fell to 49.24% 2016 but still plays the main role in the explanation of inequality. Other positive contributors to inequality in UOH in 2016 were basic insurance (12.03%), supplementary insurance (11.18%), residence (8.66%), and healthcare needs (8.37%). Alongside the economic status that explained 63.33% of the UIH inequality, education (40.61%) was another main factor behind the pro-rich distribution of UIH in 2008. Besides, economic status (218.44%) and residence (93.50%) were the main contributors to inequality in UIH 2016. The larger contribution percentage in 2016 arose from the lower inequality of UOH in this year.

**Fig. 1:**
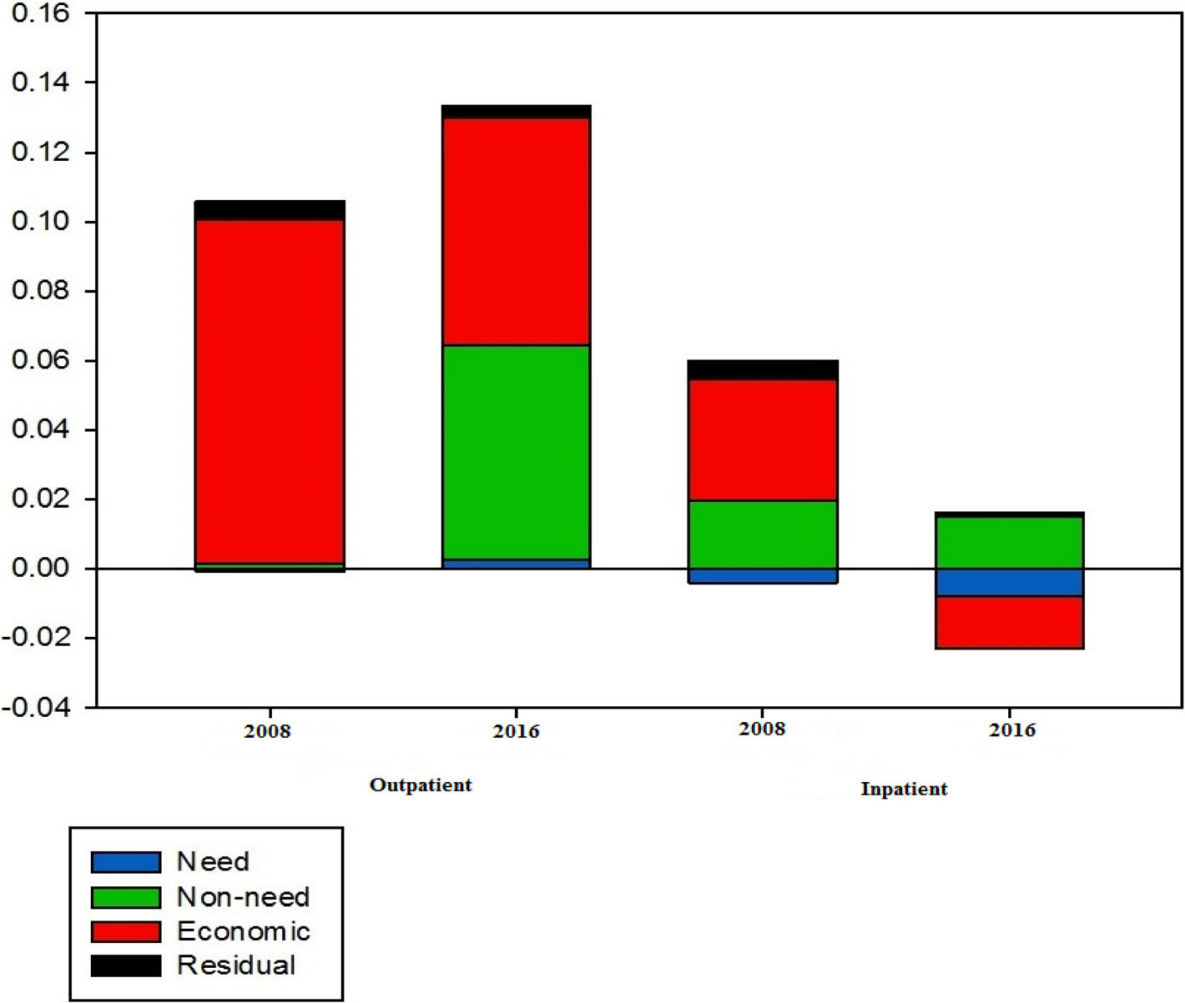
Absolute contributions of variables to the inequality in outpatient and inpatient healthcare utilization in 2008 and 2016

The absolute contributions of determinants of UOH and UIH in different periods were categorized into need, non-need, economic, and residual, as shown in Fig. 1. As observed in Fig. 1, non-need factors in all decomposition analyses operated in the pro-rich distribution of UOH and UIH. Additionally, economic status operated in a pro-poor direction for UIH only in 2016. After subtracting the absolute contribution of need factors, it is demonstrated that the degree of horizontal inequity for UOH increased from 0.106 in 2008 up to 0.131 in 2016. On the other hand, the value of this index for UIH decreased from 0.060 in 2008 to 0.001 in 2016.

**Fig. 2:**
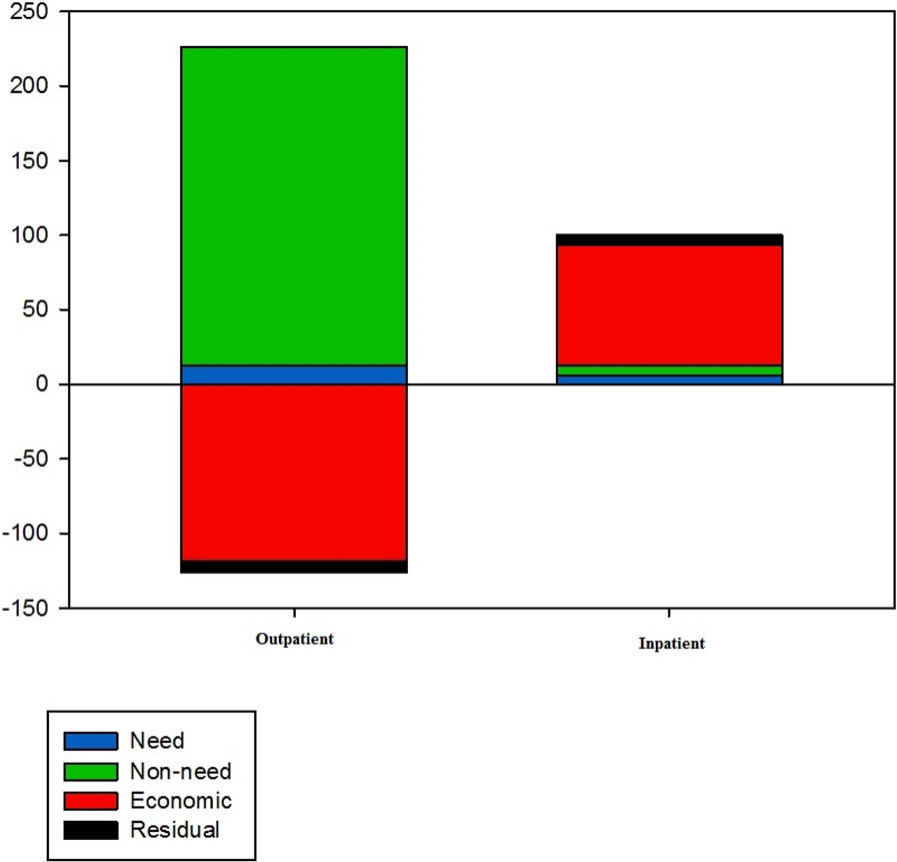
Percentage contributions of variables to the change of inequality in outpatient and inpatient healthcare utilization in Iran

The result of Oaxaca decomposition is also summarized in Table 3, Table 4, and Fig. 2. In the Oaxaca decomposition, the absolute contribution for each explanatory variable arose from two components: (1) directly, in coefficients weighted by its inequality in 2016, and (2) indirectly, through the change in inequality weighted by its coefficient effect (β) in 2008. Regarding UOH, it is demonstrated that the residence (72.88%) and supplementary insurance (65.13%) were the major factors that increase the inequality in UOH. While the change in the inequality and change in the coefficient of rural participants reinforced each other, the values for supplementary insurance operated in different directions.

Basic insurance was another main contributor to increased inequality in the UOH, which its positive contribution originated from Iranian insurance (56.88%). Surprisingly, economic status negatively contributed to this increased inequality. In terms of UIH, economic status with more than 80% contribution played the main role in the reduced inequality. The absolute contribution of this factor dominantly resulted from the change in its coefficient. Education (21.68%) and residence (13.43%) were other main contributors that could explain the reduced UIH inequality. Alongside basic insurance, supplementary insurance was negatively contributed to the reduced inequality. The negative contribution of basic insurance was rooted in Iranian health insurance that changed its inequality, and its coefficient operated contrary to the reduced inequality in UIH. As can be seen in Fig. 2, while non-need factors explained most changes in inequality in UOH, economic status was the dominant impetus for change in UIH inequality.

## Discussion

The equitable utilization of healthcare services in each health system plays a pivotal role in improving health outcomes [3, 6]. Identifying and tackling the socioeconomic inequality of healthcare utilization could help policymakers adopt tailored policies to reduce such inequalities. The present study was designed to determine the socio-economic inequality in UOH and UIH in the health system of Iran over time. Covering two time periods before and after the HTP, our research implicitly could provide some evidence about the effectiveness of the distributional desires of this program.

The results of this study showed that inequalities and inequities were favoring the rich in UOH, which significantly increased over time. To be exact, UOHs were mostly enjoyed by the better-off people after the HTP. Given that the HTP does not cover the outpatient sector completely and increases in the inflation rate of outpatient services [30], the affordability of UOH for worse-off people could be hindered [31]. The decomposition of UOH inequality showed that despite a decrease in the contribution of economic status in 2016, it was the main factor contributing to the inequality in UOH. This finding is consistent with other studies undertaken in Iran [16, 32, 33] and other countries [10, 33–35]. These findings suggest that tackling inequality in UOH is highly sensitive to economic status. In other words, if incomes were equally distributed among different wealth quintiles before and after the HTP, inequality in UOH would decrease by 94 and 49%, respectively. Therefore, it is recommended to prioritize and rearrange policies focusing on improving timely, geographical and financial access to outpatient care for the lower socio-economic groups.

Furthermore, while the educational status and the basic health insurance were the following contributing factors to inequalities in UOH in 2006, both basic and complementary health insurance and educational status were placed next in UOH in 2016. This finding is in line with the prior studies that accounted for education [36–38] or insurance [37, 39] as important drivers of inequality in healthcare utilization. OD showed that the bulk of the increase in explained inequality in UOH was due to the changes in residence in rural areas and supplementary insurance. Despite the increase in the coverage of basic insurance schemes, especially Iranian health insurance, it seems that these schemes could not provide enough affordability for the utilization of healthcare services. Thus, supplementary insurance became a dominant factor in the utilization of these services. Another study [39] also showed that after the healthcare reform, insurance plays a critical role in the increased inequality in the level of healthcare utilization in China. Moreover, although rural residents in Iran benefit from the well-established referral system, it seems that they still face remarkable obstacles to reach sophisticated healthcare services [8]. Hence, facilitating the access of rural residents to required healthcare services and enriching benefit packages of basic insurance could be recommended as effective schemes to tackle inequality in the outpatient sector of the Iranian health system.

Our results revealed that inequality in UIH not only decreased over time but also was changed from positive to negative values, showing that alongside the reduction in UIH inequality, the poor utilized inpatient services. Previous studies in other countries, however, have reported different results. While some nations could tackle income inequality in the inpatient sector [37, 40], others reported persistent inequality in this sector [41, 42]. In both surveys, the largest contribution to inequality in UIH was the economic status. Previous studies that were conducted prior to the HTP [43] and at the beginning of this policy [44] have confirmed that wealthy people have greater UIH than their poor counterparts. In this study, OD showed that the economic status was the main contributing factor that reduced inequality in the UIH. This seems to be justifiable as the provision of subsidized inpatient healthcare services through implementing the HTP could increase the utilization of these services by disadvantaged groups. In the previous study conducted in Turkey after the formulation of the Turkish HTP, the dominant contributors of pro-poor inpatient utilization were Green Card holders. Green Card is an insurance scheme that provides subsidized healthcare services for disadvantaged groups in Turkey [37]. Hence, the engagement of the public sector in providing healthcare services could facilitate access and tackle much more of socio-economic inequalities in the utilization of healthcare services. However, policymakers must ensure the sustainability of these interventions.

Within the scope of the health system, the potential effects of outside factors on the health outcomes must not be neglected. By experiencing a targeted subsidies plan in Iran [45], the income distribution dramatically changed in the country. It is believed that targeted distributional goals of this policy deteriorated in these years, and accordingly, the income inequality increased. In this situation, the health outcomes and their equities can shrink [46]. Moreover, there were huge international sanctions that not only directly decreased economic growth but also indirectly posed some obstacles for the health system, which could decline health outcomes [47].

### Strengths and limitations

This study benefits from extensive evidence of socio-economic inequalities in both UOH and UIH based on two surveys that cover HTP in Iran. This situation provides an opportunity to measure the changes in healthcare utilization over these two cross-sections. To our knowledge, this is one of the first studies conducted to measure socio-economic inequalities in the utilization of health services in Iran with some implications in relation to the HTP as the recent reform in the Iranian health system. Another strength of this study is the size of the surveys and the population-based data, which make it close to being a representative of the Iranian population. Nonetheless, some limitations must be acknowledged. First, the analysis was based on self-reported data, which is subject to reporting bias. Second, IRUHSS has no standard questions about health status, such as health-related quality of life and self-rated health. Hence, the degree of health inequality may change if suitable need variables are considered in the analysis.

## Conclusion

This study investigated the changes in socio-economic inequalities in inpatient and outpatient health care utilization over time in the health system of Iran. We revealed that inequality in UIH was reduced, while inequality in UOH increased. People with lower socio-economic status, residency in rural areas, and those with a lack of coverage of supplementary insurance had lower access to UOH. Therefore, future tailored measures should be taken to rearrange the HTP so that it covers outpatient services as well. It is also suggested that policymakers should tackle the inequalities by strengthening the benefits package of basic health insurance, extending the coverage of supplementary health insurance, and facilitating the utilization of people who live in rural areas so that UOH inequality is reduced.

## Data Availability

The datasets used during the current study were available from the corresponding author on reasonable request.

## Abbreviations

EI: Erreygers Concentration Index
HTP: Health Transformation Plan
IrUHSS: Iranian Utilization of Healthcare Services Survey
MOHME: Ministry of Health and Medical Education
PCA: Principal Component Analysis
UHC: Universal Health Coverage
UIH: Utilization of Inpatient Healthcare Services
UOH: Utilization of Outpatient Healthcare Services

## References

[1] Saito E, Gilmour S, Yoneoka D, Gautam GS, Rahman MM, Shrestha PK, Shibuya K (2016). Inequality and inequity in healthcare utilization in urban Nepal: a cross-sectional observational study. Health Policy Plan.

[2] Atun R, de Andrade LO, Almeida G, Cotlear D, Dmytraczenko T, Frenz P, Garcia P, Gomez-Dantes O, Knaul FM, Muntaner C (2015). Health-system reform and universal health coverage in Latin America. Lancet (London, England).

[3] Kim C, Saeed KMA, Salehi AS, Zeng W (2016). An equity analysis of utilization of health services in Afghanistan using a national household survey. BMC Public Health.

[4] Morton S, Pencheon D, Squires N (2017). Sustainable development goals (SDGs), and their implementation: a national global framework for health, development and equity needs a systems approach at every level. Br Med Bull.

[5] Culyer AJ, Wagstaff A (1993). Equity and equality in health and health care. J Health Econ.

[6] Zere E, Moeti M, Kirigia J, Mwase T, Kataika E (2007). Equity in health and healthcare in Malawi: analysis of trends. BMC Public Health.

[7] Homaie Rad E, Vahedi S, Teimourizad A, Esmaeilzadeh F, Hadian M, Torabi Pour A (2013). Comparison of the effects of public and private health expenditures on the health status: a panel data analysis in eastern mediterranean countries. Int J Health Policy Manag.

[8] Vahedi S, Rezapour A, Mohammadbeigi A, Khosravi A (2018). Economic inequality in outpatient healthcare utilization: the case of Iran. J Res Health Sci.

[9] Bonfrer I, van de Poel E, Grimm M, Van Doorslaer E (2014). Does the distribution of healthcare utilization match needs in Africa?. Health Policy Plan.

[10] van Doorslaer E, Koolman X, Jones AM (2004). Explaining income-related inequalities in doctor utilisation in Europe. Health Econ.

[11] Van Doorslaer E, Masseria C, Koolman X (2006). Inequalities in access to medical care by income in developed countries. CMAJ.

[12] Lu JF, Leung GM, Kwon S, Tin KY, Van Doorslaer E, O'Donnell O (2007). Horizontal equity in health care utilization evidence from three high-income Asian economies. Soc Sci Med (1982).

[13] Hosseinpoor AR, Bergen N, Schlotheuber A, Boerma T (2018). National health inequality monitoring: current challenges and opportunities. Global Health Action.

[14] Davari M, Haycox A, Walley T (2012). The Iranian health insurance system; past experiences, present challenges and future strategies. Iran J Public Health.

[15] World Bank (2008). Islamic Republic of Iran - Health sector review (Vol. 2) : Background sections.

[16] Hajizadeh M, Connelly LB, Butler JR, Khosravi A (2012). Unmet need and met unneed in health care utilisation in Iran. Int J Soc Econ.

[17] Chavehpour Y, Rashidian A, Woldemichael A, Takian A (2019). Inequality in geographical distribution of hospitals and hospital beds in densely populated metropolitan cities of Iran. BMC Health Serv Res.

[18] Doshmangir L, Bazyar M, Majdzadeh R, Takian A (2019). So near, so far: four decades of health policy reforms in Iran, achievements and challenges. Archives of Iranian medicine.

[19] Domestic general government health expenditure (GGHE-D) per capita in PPP int$ Data by country [http://apps.who.int/gho/data/node.main.GHEDGGHEDpcPPPSHA2011?lang=en].

[20] Mohammadbeigi A, Hassanzadeh J, Eshrati B, Rezaianzadeh A (2013). Socioeconomic inequity in health care utilization, Iran. J Epidemiol Global Health.

[21] Moradi-Lakeh M, Vosoogh-Moghaddam A (2015). Health sector evolution plan in Iran; equity and sustainability concerns. Int J Health Policy Manag.

[22] Ibrahimipour H, Maleki M-R, Brown R, Gohari M, Karimi I, Dehnavieh R (2011). A qualitative study of the difficulties in reaching sustainable universal health insurance coverage in Iran. Health Policy Plan.

[23] Omani-Samani R, Mansournia MA, Almasi-Hashiani A, Sepidarkish M, Safiri S, Khedmati Morasae E, Amini Rarani M (2018). Decomposition of socioeconomic inequalities in preterm deliveries in Tehran, Iran. Int J Gynaecol Obstetrics.

[24] Vyas S, Kumaranayake L (2006). Constructing socio-economic status indices: how to use principal components analysis. Health Policy Plan.

[25] Yazdi-Feyzabadi V, Rashidian A, Amini Rarani M (2019). Socio-economic inequality in unhealthy snacks consumption among adolescent students in Iran: a concentration index decomposition analysis. Public Health Nutr.

[26] Wagstaff A, Paci P, van Doorslaer E (1991). On the measurement of inequalities in health. Soc Sci Med.

[27] Erreygers G (2009). Correcting the concentration index. J Health Econ.

[28] Ramezani Doroh V, Vahedi S, Arefnezhad M, Kavosi Z, Mohammadbeigi A (2015). Decomposition of health inequality determinants in shiraz, south-West Iran. J Res Health Sci.

[29] Rizal MF, van Doorslaer E (2019). Explaining the fall of socioeconomic inequality in childhood stunting in Indonesia. SSM - Population Health.

[30] Bayati M, Sarikhani Y, Rad EH, Heydari ST, Lankarani KB: An Analytical Study on Healthcare Inflation Rate and Its Most Important Components in Iran. Shiraz E-Med J 2014;15(4). 10.17795/semj23627.

[31] Stellenberg EL (2015). Accessibility, affordability and use of health services in an urban area in South Africa. Curationis.

[32] Ranjbar Ezzatabadi M, Khosravi A, Bahrami MA, Rafiei S (2018). Socio-economic inequalities in health services utilization: a cross-sectional study. Int J Health Care Quality Assurance.

[33] Yiengprugsawan V, Carmichael G, Lim LY, Seubsman S, Sleigh A (2011). Explanation of inequality in utilization of ambulatory care before and after universal health insurance in Thailand. Health Policy Plan.

[34] Van de Poel E, Van Doorslaer E, O’Donnell O (2012). Measurement of inequity in health care with heterogeneous response of use to need. J Health Econ.

[35] Masseria C, Giannoni M (2010). Equity in access to health care in Italy: a disease-based approach. Eur J Pub Health.

[36] Debnath A, Bhattacharjee N (2018). Wealth-based inequality in child immunization in India: a decomposition approach. J Biosoc Sci.

[37] Sozmen K, Unal B (2016). Explaining inequalities in health care utilization among Turkish adults: findings from health survey 2008. Health Policy (Amsterdam, Netherlands).

[38] Mullachery P, Silver D, Macinko J (2016). Changes in health care inequity in Brazil between 2008 and 2013. Int J Equity Health.

[39] Flato H, Zhang H (2016). Inequity in level of healthcare utilization before and after universal health coverage reforms in China: evidence from household surveys in Sichuan Province. Int J Equity Health.

[40] Somkotra T (2011). Measurement and explanation of horizontal (in)equity in health care utilization among Thais after universal coverage policy implementation. Asia Pac J Public Health.

[41] Zhou Z, Gao J, Fox A, Rao K, Xu K, Xu L, Zhang Y (2011). Measuring the equity of inpatient utilization in Chinese rural areas. BMC Health Serv Res.

[42] Dorjdagva J, Batbaatar E, Dorjsuren B, Kauhanen J (2015). Income-related inequalities in health care utilization in Mongolia, 2007/2008–2012. Int J Equity Health.

[43] Etemad K, Yavari P, Mehrabi Y, Haghdoost A, Motlagh ME, Kabir MJ, Jafari N (2015). Inequality in utilization of in-patients health Services in Iran. Int J Prev Med.

[44] Abouie A, Majdzadeh R, Khabiri R, Hamedi-Shahraki S, Emami Razavi SH, Yekaninejad MS: Socioeconomic inequities in health services' utilization following the Health Transformation Plan initiative in Iran. Health Policy Plan. 2018;33(10):1065–72. 10.1093/heapol/czy096 30535054

[45] Guillaume D, Zytek R, Farzin MR (2011). Iran:the chronicles of the subsidy reform (working paper no. 11–167). In.

[46] Yahyavi Dizaj J, Anbari Z, Karyani AK, Mohammadzade Y (2019). Targeted subsidy plan and Kakwani index in Iran health system. J Educ Health Promotion.

[47] Setayesh S, Mackey TK (2016). Addressing the impact of economic sanctions on Iranian drug shortages in the joint comprehensive plan of action: promoting access to medicines and health diplomacy. Glob Health.

